# Evaluation of Electrovibration Stimulation with a Narrow Electrode

**DOI:** 10.3390/mi9100483

**Published:** 2018-09-22

**Authors:** Hiroki Ishizuka, Seiya Komurasaki, Kunihiro Kato, Hiroyuki Kajimoto

**Affiliations:** 1 Department of Intelligent Mechanical Systems Engineering, Institute of Technology, Kagawa University, 2217-20 Hayashi-cho, Takamatsu, Kagawa 761-0396, Japan; s15t421@stu.kagawa-u.ac.jp; 2 Department of Information Science and Technology, The University of Tokyo, 7-3-1 Hongo, Bunkyo, Tokyo 113-8654, Japan; kkunihir@acm.org; 3 Department of Informatics, University of Electro-Communications, 1-5-1 Chofugaoka, Chofu, Tokyo 182-8585, Japan; kajimoto@kaji-lab.jp

**Keywords:** tactile display, electrovibration, MEMS, micro-actuator

## Abstract

Recently, electrovibration tactile displays were studied and applied to several use cases by researchers. The high-resolution electrode for electrovibration stimulus will contribute to the presentation of a more realistic tactile sensation. However, the sizes of the electrodes that have been used thus far are of the millimeter-order. In this study, we evaluated whether a single narrow electrode was able to provide the electrovibration stimulus adequately. The widths of the prepared electrodes were 10, 20, 50, 100, 200, and 500 μm. We conducted a sensory experiment to characterize each electrode. The electrodes with widths of 50 μm or less were not durable or suitable for the applied signal, although the subjects perceived the stimulus. Therefore, we conducted the experiment without using these non-durable electrodes. The voltage waveform condition affected perception, and the subjects were not sensitive to the electrovibration stimulus at low frequencies. In addition, the stroke direction of the fingertip had a significant effect on perception under certain conditions. The results indicate that electrovibration stimulation requires an electrode with a width of only a few hundred micrometers for stimulation.

## 1. Introduction

Recently, tactile displays which provide tactile feedback to users have attracted the interest of both researchers and industry. Tactile displays can provide not only simple tactile stimuli, such as braille dot patterns [1,2] or vibration, but also tactile sensations such as surface textures [3,4]. Tactile displays are categorized into electrical type or mechanical type depending on the stimulation principle.

Electrical tactile displays provide tactile stimuli using current. Electrical tactile displays consist of bare electrodes that need to be contacted to the skin of users. When voltage is applied to the electrodes, current flows through the skin. Then, mechanoreceptors inside the skin are stimulated by the current and the users perceive tactile sensations such as pressure or vibration. Kajimoto et al. developed a cylindrical electrical tactile display with more than a thousand electrodes [5]. The diameter and interval of the electrodes are 2 mm and 3 mm, respectively. The electrical tactile display can provide electrical stimuli to any location on the palm. Electrical tactile displays can potentially be miniaturized and integrated into other devices since they only require electrodes which are connected to both high voltage and ground. However, the problem of these electrical tactile displays is the stability of the presented tactile sensation [6]. Another type of electrical tactile display was proposed by Deepak et al. [7]. They developed an electrical tactile display with electric arcs. This electrical tactile display can provide electrical stimuli in a non-contact state. However, the presented tactile stimuli are varied between users and not reproduced easily.

Mechanical tactile displays stimulate the surface of the skin using displacement or vibration of actuators [8,9,10,11]. Then, the tactile mechanoreceptors are stimulated and users perceive the tactile stimuli. Owing to the simplicity of the stimulation principles and the stability of the presented tactile stimuli, mechanical tactile displays are widely used. For example, Qi et al. developed a tactile display consisting of an array of piezo actuators. The stimulation resolution of the tactile display was 1.2 mm × 1.4 mm [12]. Luk et al. integrated the tactile display into an information device [13]. Schorr et al. developed a wearable tactile device with motors and link structures for virtual object manipulation and exploration [14]. Liu et al. developed a multi-finger tactile display with pneumatic actuators [15]. They applied the tactile display to virtual palpations and the presented stimuli were successfully discriminated by the subjects. Yang et al. developed a miniature tactile display which consists of an array of magnetic actuators and micro-fabricated springs [16]. Matuura et al. developed a tactile display to emulate the surfaces of woods or clothes with a voice coil motor that can provide higher frequency stimuli than normal magnetic actuators [17]. Culbertson et al. arrayed the voice coil motors and developed a wearable tactile display to provide tactile stimuli to the forearm [18]. By optimizing the actuation, the tactile display can provide continuous lateral motion.

**Figure 1:**
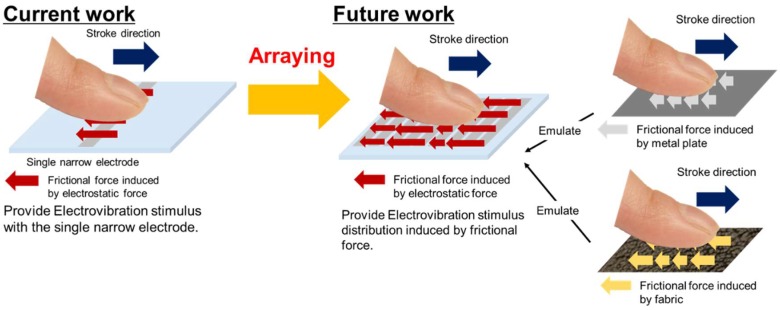
Concept of the multi-electrode electrovibration tactile display. The presented frictional force is spatially changed via voltage control, as shown on the right side. The multi-electrode electrovibration tactile display has a potential to emulate frictional force distribution of real materials. The objective of this study is the evaluation of the single narrow electrode, as shown on the left side.

Electrovibration tactile displays stimulate mechanically the contacting skin by modulating the frictional force and have a simple structure, since only an insulator layer and an electrode are required for the stimulation. The principle of electrovibration tactile displays was accidently discovered by Mallinkrodt in 1952 [19]. Electrovibration tactile displays have been studied for several decades [20,21,22,23]. For example, Bau et al. integrated an electrovibration tactile display into a touch screen display and developed an information device [24]. Vardar et al. evaluated the fundamental characteristics of an electrovibration tactile display, such as the relationship between the frequency and perception [25]. Jiao et al. optimized the signals to an electrovibration tactile display to provide more realistic tactile sensation [26]. The electrovibration tactile displays mentioned above consist of a single electrode. Therefore, constant vibration is provided to the contacting skin. When a human touches the surface of an object, the contacting part of the fingertip deforms spatially [27,28]. Additionally, the textures of day-to-day surfaces are fine [29]. For example, wood surface has bumps with a pitch of a few micrometers. Therefore, the high-resolution electrode for electrovibration stimulus, which results in spatial and detail control of frictional force, has a possibility to emulate the textures of the day-to-day surfaces, as shown in the right side of Figure 1. The multi-electrode electrovibration tactile display consists of narrow electrodes, and the voltage to each electrode is controlled separately. The multi-electrode electrovibration tactile display can be easily applied to the existing applications, including the touch screen display [24], and has a possibility to provide more realistic tactile sensations than existing electrovibration tactile displays through applications.

There have been few studies dealing with multi-electrode electrovibration tactile displays [21,22,23,30]. However, the sizes of the evaluated electrodes were of millimeter-order and an electrode with a size of a few hundred micrometers or less has never been developed and evaluated.

To develop a multi-electrode electrovibration tactile display, we first evaluated whether a single electrode with a width of a few hundred micrometers or less was able to provide electrovibration stimuli, as shown in the left side of Figure 1. The realization of electrovibration stimuli with a narrower electrode contributes to not only the high stimuli resolution, but also the miniaturization of the tactile display. For this purpose, firstly, we fabricated an array of electrodes with widths of 10, 20, 50, 100, 200, and 500 μm. Then, we investigated whether the subjects were able to perceive the electrovibration stimulus with the narrow electrodes and determined the narrowest width that was able to be applied. In order to evaluate the perceptual trend, we evaluated the relationship between the frequency of the applied voltage and the perception of the subjects for each single electrode. The fingertip has an elliptical shape. The contact area between the fingertip and a flat surface is also elliptical [31]. Therefore, the position relationship between the fingertip and electrodes also affects the overlap area between the fingertip and the rectangular electrodes. This results in a difference in the intensity of frictional force and perception. To evaluate the effect of the positional relationship, we conducted evaluations for two stroke directions: when the fingertip is parallel to the electrode and when it is vertical to the electrode. The average threshold voltages of the two direction were compared and a paired t-test was performed to evaluate the effect of the stroke direction statistically. Finally, we discuss the optimal sizes of the narrow electrode with the obtained results.

## 2. Principle 

**Figure 2:**
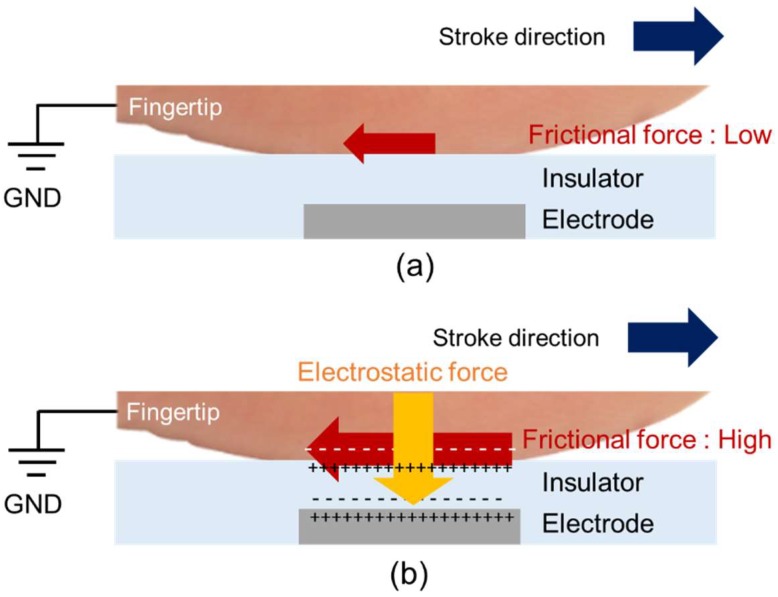
Principle of the electrovibration tactile display. (**a**) The electrovibration tactile display consists of an insulator layer and an electrode. Without an applied voltage, the frictional force is low. (**b**) With an applied voltage, the electrostatic force is high and the resulting frictional force increases because of the electrostatic force.

Figure 2 shows the principle of the electrovibration tactile display. The electrovibration tactile display in this study consists of an insulator layer and a narrow electrode. The contacting fingertip is connected to the ground, and the electrode is connected to the high voltage terminal. Without voltage, no external force is applied to the fingertip and the user perceives a smooth surface, as shown in Figure 2a. Under an external voltage, the fingertip and the electrode are oppositely charged because of the dielectric polarization of the insulator. As a result, the electrostatic force is increased between the fingertip and the electrode and the fingertip is attracted toward the electrode, as shown in Figure 2b. We hypothesize that the electrostatic force is induced to the overlap area between the fingertip and the narrow electrode and simplify the model. The induced electrostatic force and the resulting frictional force are expressed as follows [25]:

(1) $$F=\frac{A\varepsilon \varepsilon_{0}}{2}{\left(\frac{V^{\prime }\left(t\right)}{d}\right)}^{2}$$

(2) $$F^{\prime }=\mu \left(F+N\right)=\mu \left(\frac{A\varepsilon \varepsilon_{0}}{2}{\left(\frac{V^{\prime }\left(t\right)}{d}\right)}^{2}+N\right)F^{\prime }=\mu F=\mu \frac{A\varepsilon \varepsilon_{0}}{2}{\left(\frac{V^{\prime }\left(t\right)}{d}\right)}^{2}$$

where *F* is the electrostatic force which acts as the attractive force, *ε* is the relative permeability of the stratum corneum, *ε*_0_ is the vacuum permeability, *A* is the overlap area between the finger pad and the electrode, *V′*(*t*) is the applied voltage across the stratum corneum, *d* is the thickness of the stratum corneum, *F′* is the resulting frictional force, and *μ* is the frictional coefficient. The waveform of the voltage across the stratum corneum is different from the waveform of the applied voltage since the applied voltage is filtered by the equivalent circuit formed by the body and the electrovibration tactile display [25]. Therefore, the waveform of the frictional force is different from the waveform of the applied voltage. Normally, an applied voltage to an electrovibration tactile display is periodically changed. The resulting frictional force is also periodically changed and the user perceives a vibrational sensation. In this study, we designed the electrodes with widths of 10, 20, 50, 100, 200 μm, and 500 μm to determine the optimal width of a single electrode. The length of the electrodes was 15 mm. The designed thickness of the insulator was 8 μm. Two sets of electrodes were formed on a glass substrate.

## 3. Fabrication Process

**Figure 3:**
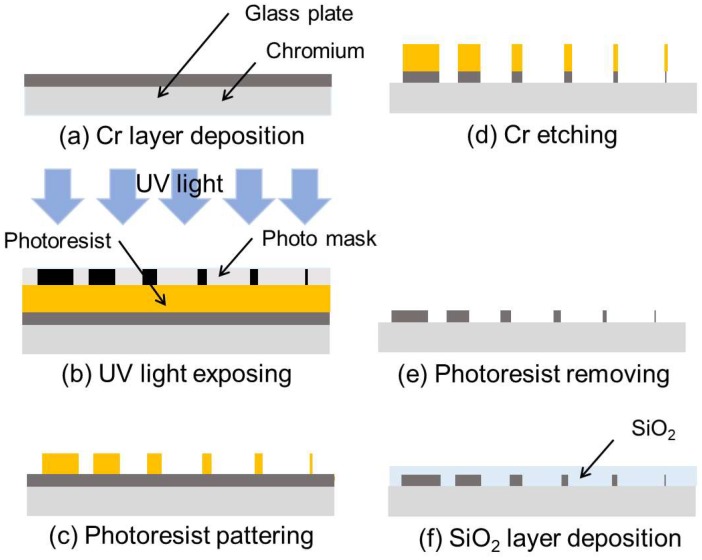
Fabrication process of the array of the electrodes. (**a**) Cr layer deposition. (**b**) UV light exposure. (**c**) Photoresist patterning. (**d**) Cr etching. (**e**) Photoresist removing. (**f**) SiO_2_ layer deposition.

**Figure 4:**
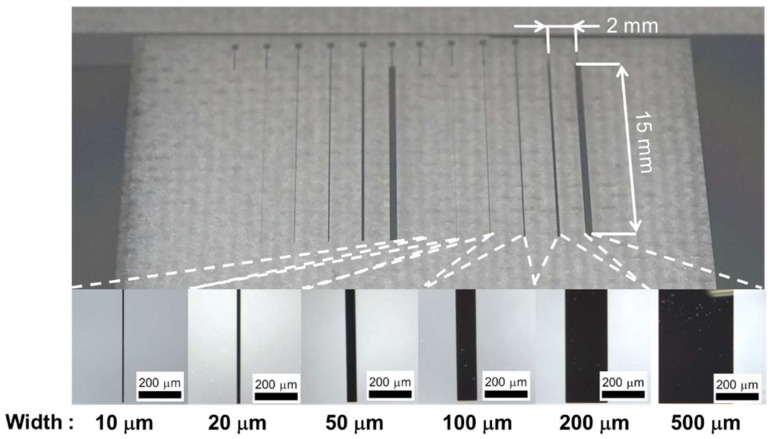
Images of the fabricated array of the electrodes. Two sets of the electrodes with widths of 10, 20, 50, 100, 200, and 500 μm were formed on a glass substrate. The designed pitch of the electrodes was 2 mm. The length of the electrodes was 15 mm.

Figure 3 shows the fabrication process of the array of the electrodes for the electrovibration tactile display. A glass substrate was dipped into hydrolysis with sulfuric acid to remove organic matter. Cr was deposited on the glass plate for 7 min with the sputtering process, as shown in Figure 3a. The resulting thickness of the Cr layer was 100 nm. A positive photoresist was deposited on the Cr layer by spin-coating at 3000 rpm. The photoresist was exposed to UV light with a photo mask, as shown in Figure 3b. Then, the photoresist was selectively dissolved with a photoresist developer, and the patterned photoresist was obtained, as shown in Figure 3c. The bared part of the Cr layer was dissolved by a Cr etching solution and an electrode pattern was obtained, as shown in Figure 3d. The remaining photoresist layer was dissolved with hydrolysis with sulfuric acid, as shown in Figure 3e. SiO_2_ was deposited on the glass plate with the sputtering process for 13 h. An insulator layer with a thickness of 8 μm was formed on the electrode pattern, as shown in Figure 3f. The fabricated array of electrodes is shown in Figure 4. The six electrodes were arranged with a pitch of 2 mm. Two sets of six electrodes are formed on a substrate. We fabricated a total of six arrays. The average widths of the electrodes were 8.5, 19.1, 50.4, 102.1, 207.6, and 513.0 μm. The average thickness of the insulator layer was 8.9 μm. The glass plate was fixed on a printed board and each electrode was connected to wires using wire bonding. Finally, the surface was coated with liquid fluorine resin (Fusso, API Corp., Tokyo, Japan) to reduce frictional force.

## 4. Experimental Procedure

We conducted a sensory experiment with eight subjects (seven males and one female; average age: 22.3 years, SD: 0.8). This experiment was approved by the Research Ethics Committee of Kagawa University (29-002). In this experiment, we investigated whether a single electrode with a width of a few hundred mm or less was able to provide an electrovibration stimulus. The electrode conditions, such as the applied voltage or position to the stroke direction, were also investigated to characterize the narrow electrodes.

**Figure 5:**
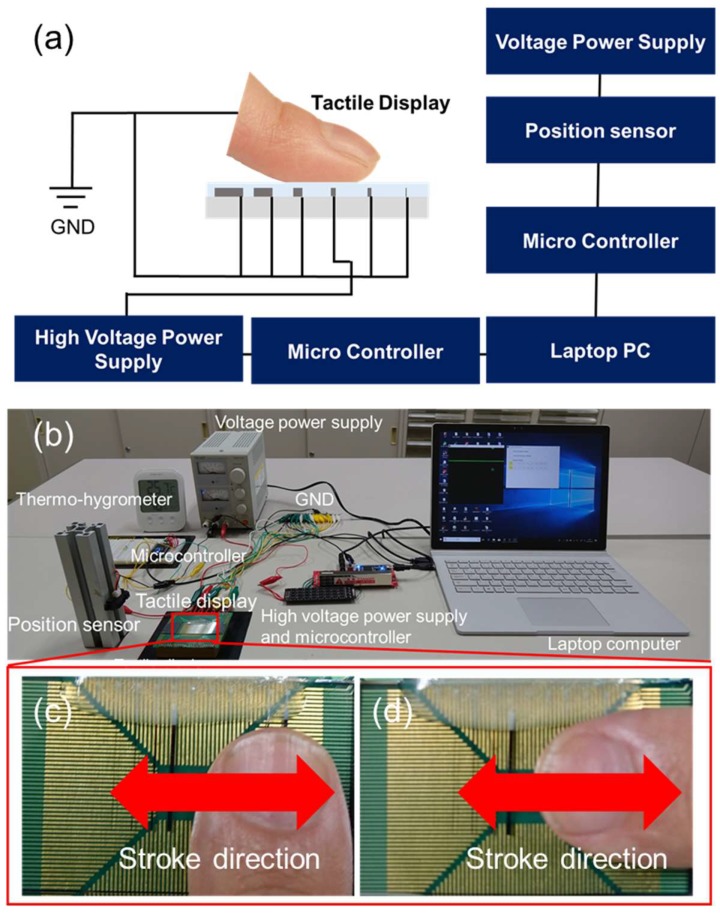
(**a**) Schematic illustration of the experimental setup. The selected electrode is connected to a high voltage power supply and other electrodes are grounded. In this case, the fourth electrode from the left is connected to the high voltage and the other electrodes are connected to the ground. (**b**) Actual photograph of the experimental setup. (**c**–**d**) Images of the stroke directions.

Figure 5a shows the schematic illustration of the experimental setup. The experimental setup consisted of a laptop computer (Surface Pro, Microsoft Corp., Redmond, WA, USA), microcontrollers (mbed LPC 1768, ARM Ltd, Cambridge, UK), a fabricated tactile display, a high voltage power supply (MHV12-1.0k, Bellnix Co., Ltd, Saitama, Japan), a position sensor (GP2Y0A21YK, Sharp Corp., Osaka, Japan) and a power supply for the position sensor (PR18-1.2A, TEXIO Technology Corp., Kanagawa, Japan). The image of the experimental setup is shown in Figure 5b. A thermo-hygrometer (O-230, dretec Corp., Saitama, Japan) was placed to measure temperature and humidity. The value of the applied voltage was adjusted with an evaluation system on the laptop computer and the control signal was sent to the high voltage power supply through the microcontroller, which was used as a D/A converter. The applied voltage to each electrode was a pulse wave. The duty ratio of the applied voltage was 10% to avoid breakdown of the insulator layer [30]. It was possible to vary the peak voltage from 0 V to 600 V with the scroll bar of the evaluation system. The frequency of the applied voltage was selected using the evaluation system. The position of the fingertip was measured with a position sensor and sent to the laptop computer through the microcontroller, which was used as an A/D converter. The stroke speed was calculated with the measured position and displayed on the laptop computer. Before the experiment, the subjects cleaned their hands with soap. The temperature and humidity of the room were observed by the thermo-hygrometer and controlled by an air conditioner. The average room temperature was 21.2 °C and the average humidity was 45.1%.

In the experiment, only the selected electrode on the electrovibration tactile display was connected to the high voltage of the power supply while the other electrodes were connected to the ground. The width of the activated electrode was selected from 10, 20, 50, 100, 200, and 500 μm. The frequency of the applied voltage was randomly selected from 20, 40, 80, 160, 320, and 640 Hz. First, we requested the subjects to place the fingertip parallel to the width of the electrodes, as shown in Figure 5c. The subjects stroked the electrovibration tactile display with their dominant index finger and detected the activated electrode while adjusting the scroll bar with another finger. There, the subjects determined the minimal voltage at which they were able to perceive the electrovibration stimulus. The electrodes which had a lower threshold voltage had a wider voltage range for the electrovibration stimulation. This indicates that electrodes with a lower threshold voltage are better for electrovibration stimulation. Stroke speed was displayed on the laptop computer and the subjects were requested to make the stroke speed 20 mm/s, which was determined from the related study [25]. After the trial, another frequency was selected and another trial was conducted. Each frequency was selected twice and two threshold voltages were obtained under each frequency condition. A total of 12 trials were conducted for each electrode. After the series of trials, the surface of the electrovibration tactile display was cleaned with a non-woven fabric and the subjects cleaned their fingers with ethanol. Then, another electrode was selected and the threshold voltages were measured under each frequency condition. The trials were conducted for all electrodes. A total of 72 trials were conducted for the fingertip position shown in Figure 5c. The same trials were also conducted for the fingertip position shown in Figure 5d. The experiment consisted of a total of 144 trials for a subject.

## 5. Results and Discussions

**Figure 6:**
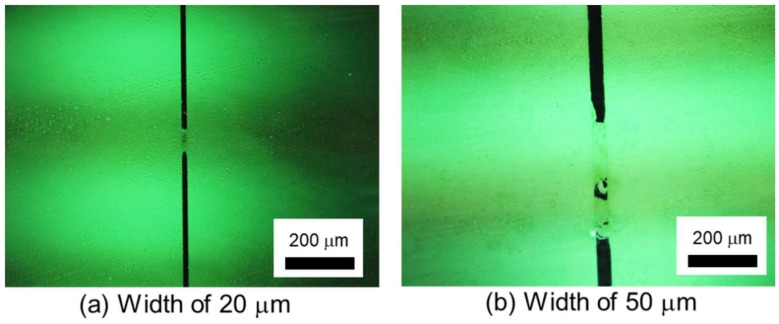
Images of the damaged electrodes. (**a**) The electrode with a width of 20 μm. (**b**) The electrode with a width of 50 μm.

In the trial experiment, the entire evaluation was not performed using the electrodes with the width of 50 μm or less. We observed the electrodes with a microscope and confirmed that the electrodes were cracked, as shown in Figure 6. The reason for the crack is the burning of the electrodes. Since the resistance of the insulator layer was high, only low current flows through the insulator layer. The narrower electrodes were not able to endure the heat induced by the minimal current at which the subjects could perceive the electrovibration stimulus. Two subjects were able to perceive the electrovibration stimulus with the electrodes having a width of 50 μm or less before the burning of the electrode. With the electrode having a width of 50 μm, the threshold of peak voltage for a subject was 200 V at 20 Hz, 160 V at 40 Hz, 136 V at 80 Hz, 200 V at 160 Hz, 270 V at 320 Hz, and 179 V at 640 Hz. To avoid the burning of electrodes, we conducted the experiment using electrodes with a width of 100 μm or more.

**Figure 7:**
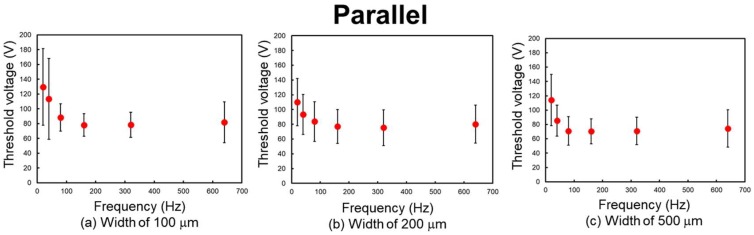
Relationship between frequency and threshold voltage. The finger was positioned parallel to the length of the electrodes, as shown in Figure 5c. (**a**) Width of 100 μm. (**b**) Width of 200 μm. (**c**) Width of 500 μm.

**Figure 8:**
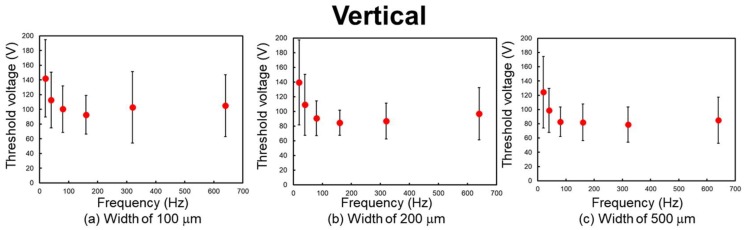
Relationship between frequency and threshold voltage. The finger was positioned vertically to the length of the electrodes, as shown in Figure 5d. (**a**) Width of 100 μm. (**b**) Width of 200 μm. (**c**) Width of 500 μm.

Figure 7, Figure 8, and Table 1 show the experimental results. The threshold peak voltage of each electrode is related to the frequency of the applied voltage. The threshold voltage increases with a decrease in frequency. This means that the subjects are more sensitive to the stimulus at high frequency. The considerable reason for this trend is considered to be the attenuation of the applied voltage. Vardar et al. reported that under low-frequency conditions, the waveform of the applied voltage is attenuated by the skin and the electrovibration tactile display [25]. Therefore, the resulting frictional force was decreased at low frequency and a high threshold voltage was required. The threshold voltage was almost constant at 80 Hz or higher. Previous studies have revealed that the tactile perception to electrovibration is almost constant at high frequency [24,25,30]. We also confirm the same trend, although the size of the electrode was narrower than that of the previous studies.

The differences in the threshold voltage were not largely affected by the difference in the width of the electrode under the same stroke direction. This indicates that these electrodes can provide almost the same intensity range of the electrovibration stimulus. According to Equation (2), the overlap area between the fingertip and the electrode determines the resulting frictional force. When the width of the electrode, which is related to the contact area, is made half or less, twice or more voltage is required to induce the same intensity of frictional force. From the obtained results, it can be seen that the threshold voltage was not made twice or five times that of the reference, when the width of the electrode was made half or one-fifth. These results indicate that the difference in the total frictional force induced by the electrostatic force to the overlap areas which have submillimeter size difference is not discriminated by humans. In contrast, the frictional force per contact area between the fingertip and the electrode is not affected by the size of the electrode. Therefore, we consider that frictional force per contact area might be more important than the total frictional force to perceive the electrovibration stimulus to the narrow area of the contacting skin.

The threshold voltage was on average 13.3 V lower when the finger was positioned parallel to the electrodes. To evaluate whether the stroke directions affected perception, we analyzed the obtained threshold voltages via a paired t-test. The obtained results and the results of the paired t-test are shown in Table 1. The results of the analysis indicate that the threshold voltage of the parallel position is significantly lower than that of the vertical position for electrodes with a width of 500 μm at 40 Hz (t(15) = 2.5147, *p* < 0.05), 500 μm at 80 Hz (t(15) = 2.6703, *p* < 0.05), 200 μm at 640 Hz (t(15) = 2.7955, *p* < 0.05), and 100 μm at 640 Hz (t(15) = 3.2103, *p* < 0.05). The results of the analysis indicate that for the parallel position, the fingertip is more sensitive to the wide electrode at low frequency and the narrow electrode at high frequency. In the case of these stroke directions, the difference in the overlap length was a few mm and provided a perceivable difference in the frictional force under the mentioned conditions. These results indicate the possibility that the difference in the frictional force to the overlap areas which have millimeter size difference can be discriminated by humans under certain conditions.

## 6. Conclusions

In this study, we fabricated an array of electrodes with widths of 500 μm or less to evaluate whether a single narrow electrode could provide an electrovibration stimulus to the subjects. A single electrode with a width of 100 μm or higher was durable to the applied voltage and successfully provided the electrovibration stimulus to the subjects. The sensitivity of the subjects to the electrovibration stimulus was related to the frequency of the electrovibration stimulus and high peak voltage was required at low frequency. The obtained trends are similar to those of previous electrovibration tactile displays with a single electrode. Additionally, the width of the electrode did not show a large effect on the perception. Moreover, the stroke direction to the electrode affected the perception under several stimulus conditions. The obtained results indicate that a single electrode with a width of a few hundred micrometers is capable of providing an electrovibration stimulus and has a potential to be applied in a small tactile display. Interestingly, electrodes with a width of 50 μm or less also showed the potential to provide electrovibration stimuli; however, voltage or current control is needed to avoid the burning of the electrodes. Additionally, the thicker electrode design is also effective to prevent the burning. The obtained results are effective for the design and fabrication of small or multi-electrode electrovibration tactile displays. In this study, we only revealed the characteristics of the narrower electrodes and did not demonstrate the concept of the multi-electrode electrovibration tactile display. In the next study, to demonstrate the concept, we plan to design and fabricate an array of 15 electrodes with a width of 100 μm and a length of 15 mm. The expected size of the multi-electrode electrovibration tactile display is almost 15 mm × 15 mm. Then, we will evaluate the fundamental characteristics, such as two-electrode discrimination, and how subjects perceive electrovibration stimuli induced by a frictional force distribution presented through the electrovibration tactile display, and consider the tactile rendering method for the multi-electrode electrovibration tactile display.

## Figures and Tables

**Table 1:** Summary of the experimental results.

Width (μm)	Frequency (Hz)	Parallel		Vertical		*p*-Value
		Average Voltage (V)	SD (V)	Average Voltage (V)	SD (V)	
100	20	129.7	52.0	142.3	52.7	0.49
100	40	113.6	54.5	112.8	37.8	0.96
100	80	88.5	18.4	100.4	31.7	0.20
100	160	78.1	15.3	92.8	26.2	0.13
100	320	78.6	16.9	103.0	48.6	0.06
100	640	81.9	27.8	105.1	41.9	**0.01**
200	20	110.1	32.0	139.5	57.8	0.06
200	40	93.3	27.1	109.1	41.4	0.14
200	80	83.8	26.9	90.9	23.6	0.27
200	160	77.1	22.8	84.6	17.1	0.13
200	320	75.4	24.2	87.0	24.3	0.08
200	640	80.3	25.6	97.0	35.7	**0.03**
500	20	114.1	35.9	124.3	50.1	0.43
500	40	85.4	21.7	98.7	30.9	**0.02**
500	80	70.9	19.8	82.3	20.8	**0.02**
500	160	70.4	17.3	82.0	25.6	0.10
500	320	70.9	19.2	78.7	24.6	0.24
500	640	74.3	26.1	84.3	32.4	0.14

## References

[1] Zhao F., Fukuyama K., Sawada H. Compact Braille display using SMA wire array. Proceedings of the RO-MAN 2009—The 18th IEEE International Symposium on Robot and Human Interactive Communication.

[2] Kato Y., Sekitani T., Takamiya M., Doi M., Asaka K., Sakurai T., Someya T. (2007). Sheet-type braille displays by integrating organic field-effect transistors and polymeric actuators. IEEE Trans. Electron Devices.

[3] Kawazoe M., Kosemura Y., Miki N. (2017). Encoding and presentation of surface textures using a mechanotactile display. Sensors Actuators A Phys..

[4] Bochereau S., Sinclair S., Hayward V. (2018). Perceptual constancy in the reproduction of virtual tactile textures with surface displays. ACM Trans. Appl. Percept..

[5] Kajimoto H. Design of cylindrical whole-hand haptic interface using electrocutaneous display. Proceedings of the EuroHaptics 2012.

[6] Kajimoto H. (2012). Electrotactile display with real-time impedance feedback using pulse width modulation. IEEE Trans. Haptics.

[7] Deepak D.S., Sahoo R., Subramanian S. Sparkle: Hover feedback with touchable electric arcs. Proceedings of the 2017 CHI Conference on Human Factors in Computing Systems.

[8] Takasaki M., Kotani H., Mizuno T., Nara T. Two-dimensional active type surface acoustic wave tactile display on a computer screen. Proceedings of the 14th Symposium on Haptic Interfaces for Virtual Environment and Teleoperator Systems.

[9] Matsunaga T., Totsu K., Esashi M., Haga Y. (2013). Tactile display using shape memory alloy micro-coil actuator and magnetic latch mechanism. Displays.

[10] Gallo S., Son C., Lee H.J., Bleuler H., Cho I.J. (2015). A flexible multimodal tactile display for delivering shape and material information. Sensors Actuators A Phys..

[11] Fujii Y., Okamoto S., Yamada Y. (2016). Friction model of fingertip sliding over wavy surface for friction-variable tactile feedback panel. Adv. Robot..

[12] Lévesque V., Hayward V. Tactile graphics rendering using three laterotactile drawing primitives. Proceedings of the 2008 Symposium on Haptic Interfaces for Virtual Environment and Teleoperator Systems.

[13] Luk J., Pasquero J., Little S., MacLean K., Levesque V., Hayward V. A role for haptics in mobile interaction. Proceedings of the SIGCHI Conference on Human Factors in Computing Systems.

[14] Schorr S.B., Okamura A.M. Fingertip tactile devices for virtual object manipulation and exploration. Proceedings of the 2017 CHI Conference on Human Factors in Computing Systems.

[15] Li M., Luo S., Nanayakkara T., Seneviratne L.D., Dasgupta P., Althoefer K. (2014). Multi-fingered haptic palpation using pneumatic feedback actuators. Sens. Actuators A Phys..

[16] Yang T.H., Kim S.Y., Kim C.H., Kwon D.S., Book W.J. Development of a miniature pin-array tactile module using elastic and electromagnetic force for mobile devices. Proceedings of the 2009 World Haptics Conference.

[17] Matsuura Y., Okamoto S., Asano S., Nagano H., Yamada Y. A method for altering vibrotactile textures based on specified materials. Proceedings of the 21st IEEE International Symposium on Robot and Human Interactive Communication.

[18] Culbertson H., Nunez C.M., Israr A., Lau F., Abnousi F., Okamura A.M. A social haptic device to create continuous lateral motion using sequential normal indentation. Proceedings of the 2018 IEEE Haptics Symposium (HAPTICS).

[19] Mallinckrodt E., Hughes A.L., Sleator W. (1952). Perception by the Skin of Electrically Induced Vibrations. Science.

[20] Strong R.M., Troxel D.E. (1970). An Electrotactile Display. IEEE Trans. Man Mach. Syst..

[21] Grimnes S. (1983). Electrovibration, cutaneous sensation of microampere current. Acta Physiol. Scand..

[22] Tang H., Beebe D.J. (1998). A microfabricated electrostatic haptic display for persons with visual impairments. IEEE Trans. Rehabil. Eng..

[23] Yamamoto A., Nagasawa S., Yamamoto H., Higuchi T. (2006). Electrostatic tactile display with thin film slider and its application to tactile telepresentation systems. IEEE Trans. Vis. Comput. Graph..

[24] Bau O., Poupyrev I., Israr A., Harrison C. TeslaTouch: electrovibration for touch surfaces. Proceedings of the 23nd annual ACM symposium on User interface software and technology.

[25] Vardar Y., Guclu B., Basdogan C. (2017). Effect of waveform on tactile perception by electrovibration displayed on touch screens. IEEE Trans. Haptics.

[26] Jiao J., Member S., Zhang Y., Member S., Wang D., Member S., Visell Y., Cao D., Guo X., Sun X. Data-driven rendering of fabric textures on electrostatic tactile displays. Proceedings of the 2018 IEEE Haptics Symposium (HAPTICS).

[27] Hayward V., Terekhov A.V., Wong S.-C., Geborek P., Bengtsson F., Jorntell H. (2014). Spatio-temporal skin strain distributions evoke low variability spike responses in cuneate neurons. J. R. Soc. Interface.

[28] Janko M., Wiertlewski M., Visell Y. (2018). Contact geometry and mechanics predict friction forces during tactile surface exploration. Sci. Rep..

[29] Ding S., Pan Y., Tong M., Zhao X. (2017). Tactile perception of roughness and hardness to discriminate materials by friction-Induced vibration. Sensors.

[30] Ishizuka H., Suzuki K., Terao K., Takao H., Shimokawa F., Kajimoto H. (2017). Evaluation of Multi-Electrode Effects on Electrovibration Tactile Stimulation. Trans. Jpn. Inst. Electron. Packag..

[31] Delhaye B., Barrea A., Edin B.B., Lefèvre P., Thonnard J.-L. (2016). Surface strain measurements of fingertip skin under shearing. J. R. Soc. Interface.

